# Which is best method for instillation of topical therapy to the upper urinary tract? An in vivo porcine study to evaluate three delivery methods

**DOI:** 10.1590/S1677-5538.IBJU.2016.0258

**Published:** 2017

**Authors:** Zhenbang Liu, Junxiang Ng, Arianto Yuwono, Yadong Lu, Yung Khan Tan

**Affiliations:** 1 Department of Urology, Tan Tock Seng Hospital, Singapore, TW; 2 Clinical Research Unit, Tan Tock Seng Hospital, Singapore, TW

**Keywords:** Urinary tract, carcinoma, transitional cell, adjuvant, instillation

## Abstract

**Purpose:**

To compare the staining intensity of the upper urinary tract (UUT) urothelium among three UUT delivery methods in an *in vivo* porcine model.

**Materials and methods:**

A fluorescent dye solution (indigo carmine) was delivered to the UUT via three different methods: antegrade perfusion, vesico-ureteral reflux via in-dwelling ureteric stent and retrograde perfusion via a 5F open-ended ureteral catheter. Twelve renal units were tested with 4 in each method. After a 2-hour delivery time, the renal-ureter units were harvested en bloc. Time from harvesting to analysis was also standardised to be 2 hours in each arm. Three urothelium samples of the same weight and size were taken from each of the 6 pre-defined points (upper pole, mid pole, lower pole, renal pelvis, mid ureter and distal ureter) and the amount of fluorescence was measured with a spectrometer.

**Results:**

The mean fluorescence detected at all 6 predefined points of the UUT urothelium was the highest for the retrograde method. This was statistically significant with p-value less than <0.05 at all 6 points.

**Conclusions:**

Retrograde infusion of UUT by an open ended ureteral catheter resulted in highest mean fluorescence detected at all 6 pre-defined points of the UUT urothelium compared to antegrade infusion and vesico-ureteral reflux via indwelling ureteric stents indicating retrograde method ideal for topical therapy throughout the UUT urothelium. More clinical studies are needed to demonstrate if retrograde method could lead to better clinical outcomes compared to the other two methods.

## INTRODUCTION

The gold standard for treatment of upper urinary tract urothelial carcinoma (UUT-UC) is nephroureterectomy with bladder cuff excision (1). However, with advancement in endourologic techniques, nephron-sparing treatments have been used by open, percutaneous or ureteroscopic approaches with reasonable oncologic outcomes. This is offered in low risk UUT-UC with normal contralateral kidney and also in imperative situations such as bilateral tumours, renal insufficiency or solitary kidney (2–4). Similar to adjuvant intravesical therapy for urothelial carcinoma of the bladder after trans-urethral resection, nephron sparing treatments have been paired with the instillation of adjuvant agents to the upper urinary tract (UUT) to reduce recurrence rates (5).

Three main methods have been described in the literature:

1. Antegrade perfusion via a percutaneous nephrostomy tube (6–8);
2. Intravesical administration with vesico-ureteral reflux via an indwelling ureteric stent (9–10);
3. Retrograde perfusion via an open-ended ureteric catheter (11, 12).

There is little data comparing on which of the three methods is the best. It has been shown in an ex-vivo porcine model that retrograde perfusion via an open-ended ureteral catheter results in better mean percentage of surface area stained and with higher mean staining intensities of the UUT, hence suggesting retrograde method the most efficient of the three (13). However, there is a lack of natural ureteral peristalsis, continuous urine production and intra—abdominal pressure in an ex vivo study which could have influenced these results. Hence, this study aims to compare the staining intensity of the UUT urothelium among the three UUT delivery methods in an in vivo porcine model.

## MATERIALS AND METHODS

### Experimental setup

Healthy live female pigs of the same species and weighing between 40-45kg were obtained from a local livestock company. Pigs were chosen because of their anatomic similarities to the collecting systems of humans (14). Permission was obtained from our institution for the use of the pigs and we strictly followed their ethical guidelines in this study. We carried out each of the three methods of delivering topical therapy to the UUT with 2 pigs consisting of 4 kidney-ureter units in each arm. We used indigo carmine dye as a substitute for topical therapy agents. It is known to stain the superficial layer of the urothelium and not penetrate tissue and it is a fluorescent dye. Hence the amount of fluorescence can be measured as a marker for staining intensity of the UUT urothelium. The dwell time was 2 hours in all 3 arms. After completion of the delivery method, the kidneys and ureters were harvested using open technique and sent for analysis. The time from completion of delivery method to the analysis was the same in all arms at 2 hours.

### Nephrostomy Tube technique

A flank incision was made on each side and the kidney and renal pelvis were exposed. We used a 20G intravenous plug as a substitute for nephrostomy tube. Due to technical difficulties in puncturing the calyxes in live pigs, we punctured the renal pelvis and angled the plug away from the ureter to prevent the dye solution from flowing straight down into the ureter and avoiding the calyxes. An intravenous tubing was attached to the plug and 150mLs of normal saline/indigo carmine solution was infused in by gravity using a burette to control the rate to be 1mL/min and have continuous perfusion of the UUT for 2 hours. The burette was placed 20cm above the renal pelvis. There was no leakage of dye around the plug. The rate of infusion and height was as described in earlier published data so as to maintain intra-renal pressure below 20-25cm H_2_O and mimic physiological conditions (7, 8).

### Reflux via indwelling ureteric stent technique

For the indwelling ureteric stent delivery method, a cystostomy was first made in the pig's bladder. The ureteric orifices were identified and a hydrophilic tip guidewire was inserted to the renal pelvis and then a 6F double pigtail stent was inserted in a retrograde fashion on each side. The cystostomy was closed with vicryl 3/0 sutures and a suprapubic catheter was placed. The skin and fascia were closed with sutures. The bladder was filled with 150mL of normal saline/indigo carmine solution via the suprapubic catheter and drained after 2 hours of dwell time (9, 10).

Unfortunately, there were no fluoroscopy facilities available in our centre's animal holding unit where the study was carried out to demonstrate vesico-ureteral reflux with the indwelling ureteric stents. To determine the volume required to have vesico-ureteral reflux, we first tested this in a separate pre-study using cadaveric pig kidney-ureter-bladder units. A cystostomy was made and the ureteric orifices were identified. A guidewire was inserted into the ureteric orifice and a 5F ureteric catheter was inserted to the renal pelvis. The renal collecting system was dis-tended with water through the ureteric catheter. A 20G intravenous plug was inserted into the lower pole calyx by blind technique. Position in the calyx/collecting system was confirmed with good efflux of water seen. The renal pelvis was then emptied of water and a guidewire introduced to the renal pelvis via the ureteric catheter. Indwelling 6F double pigtail ureteric stent was then inserted in retrograde method and position confirmed with palpation. The cystostomy was then closed with vicryl 3/0 sutures and the bladder filled with 100mL of water. Water was seen effluxing from the intravenous plug, demonstrating vesico-ureteral reflux with indwelling ureteric stent. A total of 4 cadaveric renal units were tested. We could not test for reflux in the in vivo study but considering the larger volume placed into the bladder (150mLs) compared to the volume demonstrated to have reflux in the cadaveric units (100mLs) and also the presence of intra-abdominal pressure in live pigs, we assumed there would be vesico-ureteral reflux in the indwelling ureteric stent arm.

### Retrograde ureteric catheter technique

For the retrograde ureteric catheter technique, a cystostomy was first made in the pig's bladder. The ureteric orifices were identified and a hydrophilic tip guidewire was inserted to the renal pelvis and then a 5F open ended ureteric catheter was inserted in a retrograde fashion on each side. The cystostomy was closed with vicryl 3/0 sutures and a suprapubic catheter was placed. The ureteric catheters were brought out to the skin and the suprapubic catheter was left open to prevent the pressure from building up as the bladder fills which could lead to iatrogenic vesico-ureteral reflux. A tubing connected the ureteric catheters to a burette and 150mL of normal saline/indigo carmine solution was infused via gravity into the nephrostomy tube technique described previously (11, 12).

### Analysis

After completion of the delivery method to the UUT, the kidneys and ureters were harvested surgically by open technique. The kidneys—ureters were removed en-bloc with a cuff of bladder taken. The time from completion of delivery method to the time of analysis was standardised to be 2 hours in all 3 arms. The 6 predefined points were upper pole, mid pole, lower pole, renal pelvis (centre portion), mid ureter (midway of the ureter length) and distal ureter (2cm from the cuff of bladder excised). Three samples of the urothelium were harvested from each of these 6 points to represent that area of the UUT and sent for analysis. The harvested urothelium were sectioned with the same dimensions and they were also weighed to confirm their uniformity. The excised tissues were then homogenized in phosphate buffered saline (PBS) with a tissue homogenizer (Omni International, GA, USA) before the fluorescence was measured using the SpectraMax M2 Microplate reader (Molecular Devices, CA, USA) with excitation and emission at 436 and 528 nm respectively.

Statistical analysis was performed using IBM SPSS Statistics and analyses of variance (ANOVA) was used to calculate the differences among the three arms means. Significance was defined as a P value <0.05.

## RESULTS

**Figure 1:**
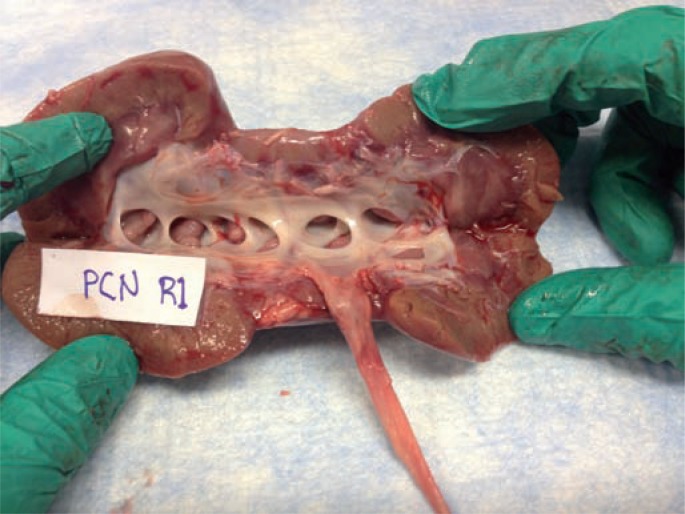
Representative image after retrograde dye delivery with ureteral catheter.

**Figure 2:**
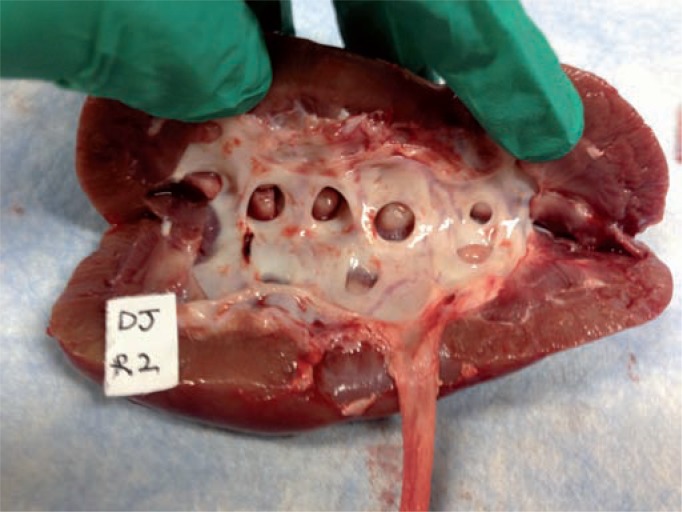
Representative image after double-pigtail stent dye delivery.

**Figure 3:**
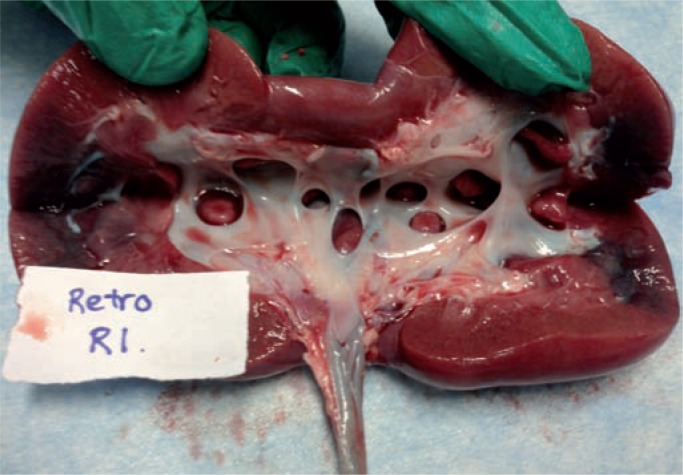
Representative image after percutaneous dye delivery.

We followed standard protocol to test 4 renal units (2 pigs) in each of the three arms. There were no complications of setup or experimentation. A sample image for stained kidney and ureter urothelium for each of the three methods are shown in Figures 1–3. Three samples of the same size and weight from each of the 6 pre-defined points of the UUT (upper pole, mid pole, lower pole, renal pelvis, mid ureter and distal ureter) were taken from each porcine unit. Results show that for the retrograde infusion of a fluorescent dye solution (indigo carmine) via an open ended ureteral catheter, the mean amount of fluorescence detected at the 6 pre-defined points was higher than the other two methods and this is significant at all the 6 points with P-value <0.05 on ANOVA analysis comparing the three methods. Comparing the antegrade perfusion method to the vesico-ureteral reflux via indwelling ureteric stent method, the mean amount of fluorescence detected was lower at all points except for the mid ureter. Results are shown in Table-1.

**Table 1:** Mean fluorescence, standard deviation and ANOVA analysis of the 3 methods at 6 pre-defined points.

Mean fluorescence (SD)	UP	MP	LP	RP	MU	DU
Antegrade	12.132 (3.646)	12.452 (3.206)	12.353 (3.106)	13.357 (2.964)	17.807 (4.513)	17.406 (2.282)
Retrograde	22.515 (3.508)	25.778 (8.089)	25.267 (3.535)	23.508 (7.482)	24.399 (8.167)	25.396 (9.394)
Reflux via DJS	15.005 (2.863)	18.791 (3.069)	18.520 (5.010)	15.604 (4.506)	14.561 (4.310)	18.005 (3.552)
ANOVA/p-value	<0.0001	<0.0001	<0.0001	<0.0001	0.001	0.004

**SD =** standard deviation; **UP =** upper pole; **MP =** mid pole; **LP =** lower pole; **RP =** renal pelvis; **MU =** mid ureter; **DU =** distal ureter; **DJS =** double-pigtail stent/ureteric stent **ANOVA =** analysis of variance

## DISCUSSION

Nephron sparing treatment options have shown good disease control similar to that of nephroureterectomy (NU) with 5 year cancer-specific survival ranging from 87-100% and 89-93% (4, 15) in low grade, non-muscle invasive UUT-UC. This is often paired with adjuvant topical therapy delivered to the UUT to improve oncological outcomes. Instillation of topical bacillus Calmette-Guerin (BCG) to the UUT has also been used as primary treatment for UUT carcinoma-in-situ (CIS). Reported case series on BCG infusion for UUT CIS is associated with uniformly high (63-100%) positive response in terms of short term normalisation of urinary cytology but with a recurrence or progression rate ranging from 0% to 50% (10, 11, 16–21).

However, results for instillation of adjuvant topical therapy to the UUT have been mixed. A non-systemic review by Rastinehad et al. (22) demonstrated efficacy of BCG in the management of upper tract CIS but no definitive efficacy of adjuvant topical therapy after endoscopic resection of Ta/T1 UUT-UC. Other centres have more encouraging results. Giannarini et al. reported their 25 year experience with antegrade infusion of BCG in curative intent for CIS and adjuvant therapy after ablative therapy in 55 patients (23). Recurrence occurred in 40% of CIS and 59% of Ta/T1 UUT-UC and progression occurred in 5% of CIS and 41% with Ta/T1 UUT-UC after a median follow-up of 42 months 11% eventually needed NU. Adverse events occurred in 20% of patients, mostly minor with one case of fatal E.coli septicaemia. Most of these patients were not medically fit for radical surgery to begin with hence the authors concluded that antegrade instillation of BCG results in high kidney preservation rate and treatment tolerability was good. Katz et al. instilled BCG and interferon-α2B in 10 patients with median age of 72 years in 11 renal units for adjuvant therapy post endoscopic ablation of UUTUC via a retrograde ureteral catheter (12). Follow-up ureteroscopy with or without biopsy was performed after a 6 week induction to evaluate response. Complete responders were placed on a maintenance regimen. With a median follow-up of 24 months, 8 patients (80%) showed a complete response to therapy and 2 had a partial response. There were no reported side effects or complications.

Several methods have been described for the instillation of topical therapy to the UUT including percutaneous nephrostomy for antegrade instillation, retrograde catheterisation and those using vesico-ureteral reflux with indwelling ureteric stents and each method has its own advantages and limitations (24). Currently, there is no consensus on which is the best method. In the first head to head comparison study between these three methods in an ex-vivo porcine model, Pollard et al. showed retrograde infusion via open ended ureteral catheter is the most efficient method of UUT therapy delivery (13). However, there are several inherent deficiencies in the ex vivo porcine model including lack of natural ureteral peristalsis, continuous urine production and the influence of intra-abdominal pressure on vesico-ureteral reflux (although the authors used a blood pressure cuff around the bladder to inflate to 8mmHG to mimic intra-abdominal pressure) which could influence the intensity of dye found in the UUT after delivery to the UUT. Hence, there was a need for an in vivo study to compare these three methods.

In this study, we aimed to compare the staining intensity of the UUT urothelium between the three UUT delivery methods in an in vivo porcine model. We measured the amount of fluorescence at 6 predefined points of the UUT with 3 samples measured at each point after instillation of the UUT with a fluorescent dye solution (indigo carmine) via the three methods of UUT delivery. For the antegrade infusion via nephrostomy arm in our study, we used an intravenous plug to access the renal pelvis instead of the calyxes for infusion of indigo carmine dye due to technical difficulties in puncturing the calyxes in the in vivo pig model. We also did not use a nephrostomy tube because antegrade insertion of a nephrostomy tube in a live pig with a non-dilated collecting system was anticipated to be very challenging and time consuming. In the ex vivo porcine study by Pollard et al., (13) retrograde insertion of nephrostomy tube via puncturing the renal pelvis with an angio-catheter and then inserting a stiff guidewire out through the renal parenchyma was found to have leakage of the dye solution around the nephrostomy tube and the risk of leakage around the defect in the collecting system created. These differences from the actual antegrade delivery method by nephrostomy tube in our study may have contributed to the lower mean amount of fluorescence detected in all the points measured compared to that of retrograde catheterisation and reflux via ureteric stent (except for the mid ureter). Other potential problems with percutaneous nephrostomy include risk of tumour seeding because of the breech in the collecting system, invasive nature and the potential to miss calyxes if the therapy solution flows straight down into the ureter. We found that with antegrade perfusion via the intravenous plug in the renal pelvis and angled away from the ureter, the renal collecting system and ureter were uniformly stained and there were no missed calyxes in our study. However, the mean fluorescence detected at the 4 points within the kidney were all lower than the 2 points in the ureter. This could be explained by the position of the plug in the renal pelvis resulting in preferential flow down the ureter despite angling it away from the ureter highlighting the possible effect of the position of the nephrostomy tube in the staining of the UUT urothelium.

The main problem with vesico-ureteral reflux via indwelling ureteric stent is that reflux is not guaranteed with the indwelling ureteric stent. Yossepowitch showed that only 59% of patients had reflux with ureteric stents (25), making this potentially an unreliable method for delivery of topical therapy to the UUT. In our study, we previously tested in cadaveric pig units, the bladder volume required to have vesico-ureteral reflux with indwelling ureteric stents as described previously. We could not test for reflux in the in vivo study but considering the larger volume placed into the bladder (150mLs) compared to the volume demonstrated to have reflux in the cadaveric units (100mLs) and also the presence of intra-abdominal pressure in live pigs, we assumed there would be reflux in the indwelling ureteric stent arm in our study. Our results show that this method stains the pre-defined points more than the antegrade perfusion arm except for the mid ureter.

Retrograde instillation of topical therapy to the UUT via open ended ureteral catheter has been published in the literature (11, 12). Pollard et al. have demonstrated in the ex vivo model that this method has advantage both in terms of area coverage and staining intensity (13). Similarly in our study, this method also resulted in the highest mean fluorescence detected at all the 6 pre-defined points and this was significant for all points on ANOVA analysis compared to the other two delivery methods stents indicating retrograde method results in the greatest staining of topical therapy throughout the UUT urothelium. More clinical studies are needed to investigate if this could correlate to better clinical outcomes by using this method compared to the other two.

One major limitation of this study was that we could not control the amount and rate of urine output among the live pigs which would dilute the dye solution. It has been shown in the in vivo pig study by Otero et al. (26) that urine output varies minute by minute but this variability was greatest under conditions of sepsis. In our study, we used healthy pigs of the same species and weight and the hydration conditions were similar so as to keep the variability of the urine output similar. The number of pigs used in each arm was also small due to financial restraints, limiting the power of our results.

## CONCLUSIONS

In this limited, initial in vivo study, we demonstrated that retrograde infusion of a fluorescent dye solution (indigo carmine) to the UUT by an open ended ureteral catheter resulted in highest mean fluorescence detected at all 6 pre-defined points of the UUT urothelium (upper pole, mid pole, lower pole, renal pelvis, mid ureter and distal ureter) compared to antegrade infusion and vesico-ureteral reflux via indwelling ureteric stents indicating retrograde method results in the greatest staining of topical therapy throughout the UUT urothelium. More clinical studies are needed to demonstrate if retrograde method could lead to better clinical outcomes compared to the other two methods.
